# Encoding of rapid time-varying information is impaired in poor readers

**DOI:** 10.1167/17.5.1

**Published:** 2017-05-01

**Authors:** Richard Johnston, Nicola J. Pitchford, Neil W. Roach, Timothy Ledgeway

**Affiliations:** School of Psychology, The University of Nottingham, University Park, Nottingham, UK; School of Psychology, The University of Nottingham, University Park, Nottingham, UK; School of Psychology, The University of Nottingham, University Park, Nottingham, UK; School of Psychology, The University of Nottingham, University Park, Nottingham, UK

**Keywords:** *poor readers*, *segmentation*, *motion perception*, *form perception*, *temporal vision*

## Abstract

A characteristic set of eye movements and fixations are made during reading, so the position of words on the retinae is constantly being updated. Effective decoding of print requires this temporal stream of visual information to be segmented or parsed into its constituent units (e.g., letters or words). Poor readers' difficulties with word recognition could arise at the point of segmenting time-varying visual information, but the mechanisms underlying this process are little understood. Here, we used random-dot displays to explore the effects of reading ability on temporal segmentation. Thirty-eight adult readers viewed test stimuli that were temporally segmented by constraining either local motions or analogous form cues to oscillate back and fourth at each of a range of rates. Participants had to discriminate these segmented patterns from comparison stimuli containing the same motion and form cues but these were temporally intermingled. Results showed that the motion and form tasks could not be performed reliably when segment duration was shorter than a temporal resolution (acuity) limit. The acuity limits for both tasks were significantly and negatively correlated with reading scores. Importantly, the minimum segment duration needed to detect the temporally segmented stimuli was longer in relatively poor readers than relatively good readers. This demonstrates that adult poor readers have difficulty segmenting temporally changing visual input particularly at short segment durations. These results are consistent with evidence suggesting that precise encoding of rapid time-varying information is impaired in developmental dyslexia.

## Introduction

Poor reading ability in adults is often associated with developmental dyslexia, especially for adults that have average or above average intelligence and do not have a history of ocular ill health, social deprivation, or other learning difficulties. Developmental dyslexia is thought to affect approximately 5%–10% of the population but there are controversies regarding how it should be defined (Siegel, 2006). Some argue that dyslexia best represents the lower end of a normal distribution of reading ability, while others suggest it is a distinct type of reading difficulty that is primarily associated with poor phonemic decoding skills (Snowling, 2000). Evidence suggests that readers with dyslexia also have a deficit processing certain types of visual information (for review, see Grinter, Maybery, & Badcock, 2010) needed for reading text. Several theories of the origin of visual impairment in dyslexia have been proposed. One of the most prominent is the dorsal stream vulnerability hypothesis (Braddick, Atkinson, & Wattam-Bell, 2003). This framework rests on the fundamental assumption that two anatomically distinct and functionally independent processing streams can be discerned in the human visual system: First, a dorsal stream projecting from primary visual cortex (V1) to parietal cortex that is thought to play a major role in tasks such as determining the global (overall) motion of objects, and second, a ventral stream projecting from V1 to the temporal lobes that has been implicated in tasks such as global form (shape) perception (Goodale & Milner, 1992; Ungerleider & Mishkin, 1982). Dorsal stream vulnerability is thought to manifest as a deficit in the processing of global motion, relative to global form.

Random-dot kinematograms (RDKs) have been used to measure dorsal stream function in a wide range of clinical populations (Grinter et al., 2010). They comprise a discrete series of images, each containing local dots that either move in the same direction on each positional update (*signal* dots) or randomly (*noise* dots). Coherence thresholds are measured and correspond to the minimum number of signal dots needed to reliably detect global motion or identify its direction (Newsome & Paré, 1988). In contrast, static global form tasks have been used to measure ventral stream function (Grinter et al., 2010). They typically consist of Glass patterns or static line segments (Glass, 1969; Hansen, Stein, Orde, Winter, & Talcott, 2001). Several studies have shown that generally poor readers and individuals who meet conventional criteria for diagnosing developmental dyslexia have significantly higher coherence thresholds than relatively good readers on RDK tasks but not on global form tasks, consistent with the dorsal stream vulnerability hypothesis (for review, see Benassi, Simonelli, Giovagnoli, & Bolzani, 2010). However, recent research has shown that coherence thresholds on RDK tasks and global form tasks are significantly and positively correlated (Braddick et al., 2016; Johnston, Pitchford, Roach, & Ledgeway, 2016a). This finding casts serious doubt on whether these psychophysical measures can be relied upon to dissociate the functional integrity of the dorsal and ventral streams. It could be indicative either of some degree of cross-talk between the two streams or a common processing stage that serves to integrate distinct object properties into a global percept (Erlikhman, Gurariy, Mruczek, & Caplovitz, 2016). Furthermore, Johnston et al. (2016a) have shown that individuals with dyslexia and generally poor readers have difficulty on global form tasks that require the integration of temporal information. Thus, a difficulty processing time-varying information could underlie the visual deficit in dyslexia rather than dorsal stream vulnerability, per se.

In reading printed text, as in processing all other forms of visual input, the visual system faces several challenges, one of which is satisfying the competing constraints of integrating local features belonging to a common object (e.g., letters in a word), while segmenting those arising from different objects (e.g., different words in a sentence; Albright & Stoner, 1995; Braddick, 1993; Nakayama, 1985). It is still unclear how this is achieved but evidence suggests that spatial segmentation might also be impaired in dyslexia. This would manifest as a difficulty in segmenting constituent components of printed text, such as letters within words and words within sentences. To explore this possibility, Cornelissen, Richardson, Mason, Fowler, and Stein (1995) gave a motion-based segmentation task to 29 adults with developmental dyslexia and an equal number of adults without developmental dyslexia. The stimuli comprised random-dot patterns that were spatially divided into horizontal segments by constraining dots in adjacent segments to move in opposing directions. Participants discriminated these segmented stimuli from uniform patterns containing dots moving in the same direction. Readers with dyslexia had significantly higher coherence thresholds than relatively good readers, suggesting that spatial segmentation may be impaired in dyslexia. However, this finding could also reflect the known deficit with global motion (integration) rather than segmentation, as the task used by Cornelissen et al. (1995) could have engendered decisions on each trial to be made by identifying the uniform stimulus. In addition, segment size remained fixed throughout the experiment despite the fact that coherence thresholds on spatial segmentation tasks depend upon segment size (Burr, McKee, & Morrone, 2006; van Doorn & Koenderink, 1982a; Watson & Eckert, 1994).

To overcome the limitations of previous research exploring spatial segmentation in dyslexia, Johnston, Pitchford, Roach, and Ledgeway (2016b) conducted an experiment with 38 adult readers who viewed random-dot displays that were specifically designed to provide a measure of object segmentation. The test stimuli were spatially divided into horizontal segments. Adjacent segments contained either local motions in opposing directions or directly analogous form cues depicting orthogonal orientations. Participants discriminated these segmented patterns from comparison stimuli containing identical motion or form cues but these were spatially intermingled. First, spatial resolution (acuity) limits were measured to determine the smallest segment size needed to reliably perform the motion and form tasks. Results showed that acuity limits were not significantly associated with scores on a composite measure of reading skill (including lexical and sublexical processing of written words). Coherence thresholds decreased as segment size increased but for the motion task, the rate of change was shallower in readers with dyslexia, and the segment size at which performance became asymptotic was larger. These findings demonstrate that spatial segmentation is also impaired in adult poor readers but only on tasks containing motion information.

To further explore why adult poor readers exhibited impaired performance on the motion-based segmentation task, Johnston et al. (2016b) devised a biologically plausible computational model. Human neuroimaging studies have shown that hMT (the human homologue of macaque V5/MT) plays a major role in the processing of global motion (Braddick et al., 2001; Tootell et al., 1995; Zeki et al., 1991). Directionally selective cells in this part of the brain operate over a range of spatial scales (Amano, Wandell, & Dumoulin, 2009) and it has been suggested that differences in receptive field size might underlie the visual deficit in developmental dyslexia and other clinical populations (Anderson et al., 2017; Chanceaux & Grainger, 2012; Grainger, Dufau, & Ziegler, 2016; Schwarzkopf, Anderson, de Haas, White, & Rees, 2014; Tydgat & Grainger, 2009). Computer simulations showed that the optimal integration field size needed to perform the motion task was one that matched the segment size. The rate of change as segment size increased was shallower, and the segment size at which performance became asymptotic was larger, when an integration zone different from the segment size was employed. This pattern of results was qualitatively similar to that found in adult poor readers, which suggests that nonoptimal sizes of integration field are employed by poor readers to pool local motion cues across space.

A discrete series of eye movements and fixations are made during reading, which means the position of words on the retinae is constantly being updated (Rayner, 1998). Hence, local visual cues must be segmented over time, as well as across space. Evidence suggests that temporal segmentation of the auditory speech stream is impaired in readers with developmental dyslexia (Goswami, 2011; Lehongre, Morillon, Giraud, & Ramus, 2013; Lehongre, Ramus, Villiermet, Schwartz, & Giraud, 2011). This raises the possibility that difficulties with temporal segmentation might represent a generic deficit in developmental dyslexia and thus should arise in other sensory modalities, such as vision.

Critical flicker fusion (CFF) thresholds can be measured to determine the maximum temporal frequency needed to segment temporally changing visual cues. To explore the relationship between CFF thresholds and reading ability, Talcott et al. (1998) asked 36 adult readers to discriminate flickering equiluminant stimuli from patterns that did not appear to flicker. Results showed that CFF thresholds were significantly correlated with scores on a standardized measure of reading ability. The temporal frequency needed to detect the flickering stimuli was lower in relatively poor readers than relatively good readers. However, Edwards et al. (2004) failed to replicate these findings using the method of adjustment to measure CFF thresholds. A disadvantage of using the method of adjustment is that it is subjective and thus, it is impossible to determine the response criterion participants used to perform the task. Furthermore, as is the case for spatial segmentation tasks, relying on a single measurement of sensitivity is not sufficient to characterize performance on temporal segmentation tasks. For example, van Doorn and Koenderink (1982b) found that coherence thresholds for detecting a temporally segmented random-dot display depend upon temporal frequency.

In summary, evidence suggests that adult poor readers and individuals with developmental dyslexia have difficulty segmenting local motion cues across space (Johnston et al., 2016b). If this reflects a generic difficulty with segmenting rapid information, difficulties with temporal segmentation may also exist in adult poor readers. Currently, the effects of reading ability on temporal segmentation are unclear, as previous research has relied on a single measurement of sensitivity and has failed to delineate temporal segmentation of motion and form information (Talcott et al., 1998). In the present study, we systematically addressed these issues by administering temporal versions of spatial segmentation tasks previously used to investigate the underlying nature of the visual deficit in dyslexia (Johnston et al., 2016b). Coherence thresholds were measured at each of a range of oscillation rates (segment durations) to measure distinct components underpinning task performance.

## Methods

### Participants

Thirty-eight adults (six men, 32 women) whose reading abilities ranged along a continuum were recruited to the study either via Student Services or an undergraduate research participation scheme at the University of Nottingham. The mean age was 20.35 years (*SD* = 2.82 months). All participants had English as their first language and were excluded from the study if they had a neurodevelopmental disorder other than developmental dyslexia, or a history of ocular ill health. Participants with a gestational age of less than 32 weeks were also excluded, as individuals born preterm typically have elevated coherence thresholds on global motion tasks (Taylor, Jakobson, Maurer, & Lewis, 2009). All participants had normal or corrected-to-normal visual acuity and gave informed consent to take part in the study according to the Declaration of Helsinki. The ethics committee at the School of Psychology, University of Nottingham, granted ethical approval for the study.

### Psychometric tests

Each participant completed tests of nonverbal intelligence (IQ) and reading ability. Nonverbal IQ was assessed using Raven's Standard Progressive Matrices (SPM; Raven, Court, & Raven, 1988). Three measures of reading ability were included to assess different components of reading skill. To measure whole-word lexical processing, the National Adult Reading Test (NART; Nelson, 1991) was administered. It comprises 50 low-frequency irregular words. The Test of Word Reading Efficiency (TOWRE), Sight Word Efficiency and Phonemic Decoding Efficiency subtests were administered to provide a standardized measure of reading ability. The TOWRE Sight Word Efficiency subtest consists of 104 regular words that vary in frequency (Torgesen, Wagner, & Rashotte, 1999). To assess sublexical decoding skills, the TOWRE Phonemic Decoding Efficiency subtest was administered. It measures speeded reading of 63 pseudo-words that vary in complexity. Participants are given 45 s to read as many words as possible in both TOWRE tests, whereas the NART is self-paced. The dependent variable for each of the three reading tests was the number of words read correctly. Summary statistics characterizing the reading abilities of the participant sample are shown in Table 1.

**Table 1:**
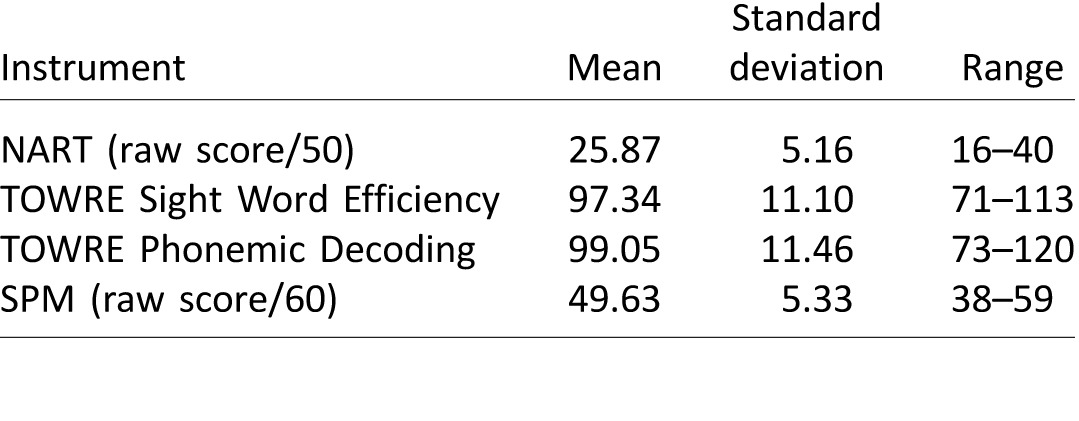
Psychometric statistics for the entire sample. *Note*: Standard scores (*M* = 100, *SD* = 15) are shown unless otherwise stated. NART = National Adult Reading Test; TOWRE = Test of Word Reading Efficiency; SPM = Raven's Standard Progressive Matrices.

### Visual stimuli

Stimuli were generated using MATLAB (MathWorks, Natick, MA) and elements of Psychtoolbox (Brainard, 1997). They were displayed on an Intergraph Interview 24hd96 monitor (frame refresh rate of 100Hz; Silicon Graphics, Inc., Milpitas, CA), which was carefully gamma-corrected using a photometer and look-up-tables. Psychophysical procedures were used to check the adequacy of the photometric gamma-correction (Ledgeway & Smith, 1994; Nishida, Ledgeway, & Edwards, 1997). The stimuli were viewed binocularly at a distance of 60 cm. They were presented within the confines of a square display window in the centre of the monitor, which subtended 7° × 7°. Each stimulus was composed of an ensemble of “black” dots (diameter 0.07°) presented against a uniform gray (34 cd/m^2^) background. The total stimulus duration in each case was 0.43 s.

### Motion task

**Figure 1:**
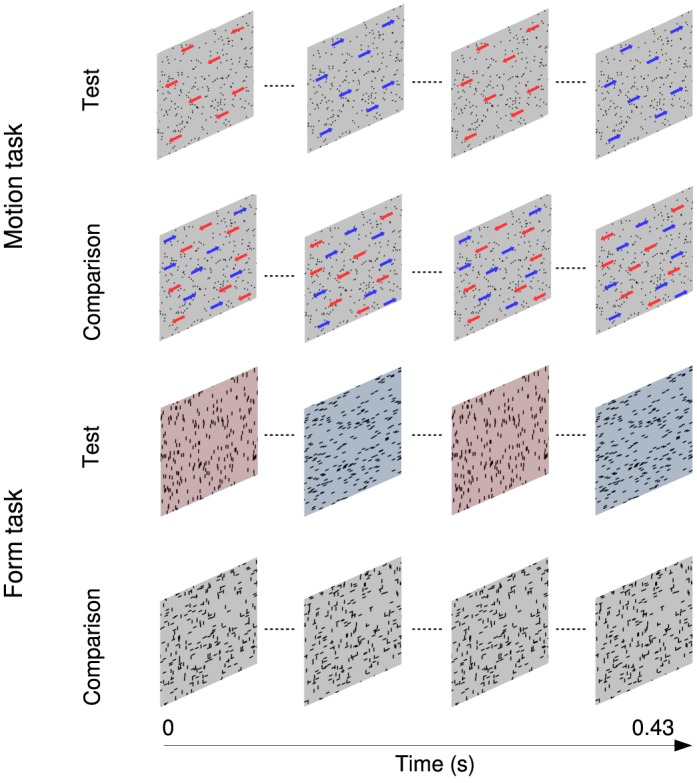
Schematic of the stimuli in the motion and form tasks. Colored overlays and directional arrows have been added for illustrative purposes only and depict how the test stimuli were temporally segmented.

The stimuli in the motion task (Figure 1) consisted of 43 images, each containing 256 dots that were presented consecutively at a rate of 100 Hz to create the perception of apparent motion. Individual dots were displaced by 0.035° on each positional update (speed = 3.5 °/s). The “strength” or coherence of the stimuli could be varied between 0% and 100% by constraining some of the dots to move in the same direction on each image update (signal dots) and others to move randomly (noise dots). Two patterns were randomly presented in succession on each trial with equal probability. They were separated by an interstimulus interval (ISI) of 0.52 s. The signal dots in the test stimulus oscillated back and forth (leftward and rightward) at each of a range of rates. Observers discriminated this temporally segmented stimulus from a comparison stimulus containing identical motion cues that were temporally intermingled. Each signal dot had a limited lifetime of 0.22 s (22 frames). At the beginning of the motion sequence it was assigned a random “age” in frames between 1 and 22. On each image update the age parameter was incremented by 1. The dot was replotted at a random location when the limit of 22 frames was exceeded.

### Form task

Stimuli in the form task (Figure 1) were generated by computing a 4-frame, random-dot motion sequence in which the signal dots were constrained to move either vertically or horizontally. The individual frames were then spatially superimposed to create a single static image (Johnston et al*.,* 2016a, 2016b; Simmers, Ledgeway, & Hess, 2005) in which the signal dots formed localized streaks, oriented (vertically or horizontally) along a common axis, while the noise dots formed random clusters. The length of each dot streak was 0.18°. The coherence of the stimuli could be varied between 0% and 100% by changing the relative proportion of signal to noise dot streaks. As per the motion task, two patterns were randomly presented in succession on each trial with equal probability. They were separated by an interstimulus interval (ISI) of 0.52 s. The dot streaks in the test stimulus oscillated back and forth (vertical and horizontal) at each of range of rates. Participants discriminated this temporally segmented stimulus from a comparison stimulus containing identical form cues that were temporally intermingled.

### Procedure

#### Temporal resolution limits

To determine the shortest segment duration (i.e., maximum oscillation rate) needed to reliably perform the motion and form task temporal resolution (acuity) limits were measured. The coherence of the stimuli was held constant at 100% in each trial block. Segment duration was varied on each trial using a two-interval, temporal forced-choice procedure and a 3-down 1-up adaptive staircase tracking the 79% correct performance level. The participants' task was to identify the temporally segmented test stimulus. The initial step size was 0.215 s and this decreased by half after each reversal. The staircase procedure terminated after 12 reversals and the arithmetic mean of the last six reversals was the acuity limit from that staircase. The reported acuity limit for each observer corresponds to the mean of at least four staircases and the order of testing was randomized across the motion and form tasks.

#### Coherence thresholds

Coherence thresholds were obtained in a similar manner to acuity limits. However, segment duration was held constant in each trial block. It ranged from 0.03–0.215 s in equal logarithmic steps. The coherence of the stimuli was varied on each trial using a 3-down 1-up adaptive staircase tracking the 79% correct performance level and the participants' task was to identify the temporally segmented test stimulus. The initial step size was equal to the total number of elements in the display and this decreased by half after each reversal. The staircase procedure terminated after 12 reversals and the arithmetic mean of the last six reversals was the coherence threshold from that staircase. The reported coherence threshold for each observer at a given segment duration corresponds to the mean of at least four staircases and the order of testing was randomized across the motion and form tasks.

#### Curve fitting

**Figure 2:**
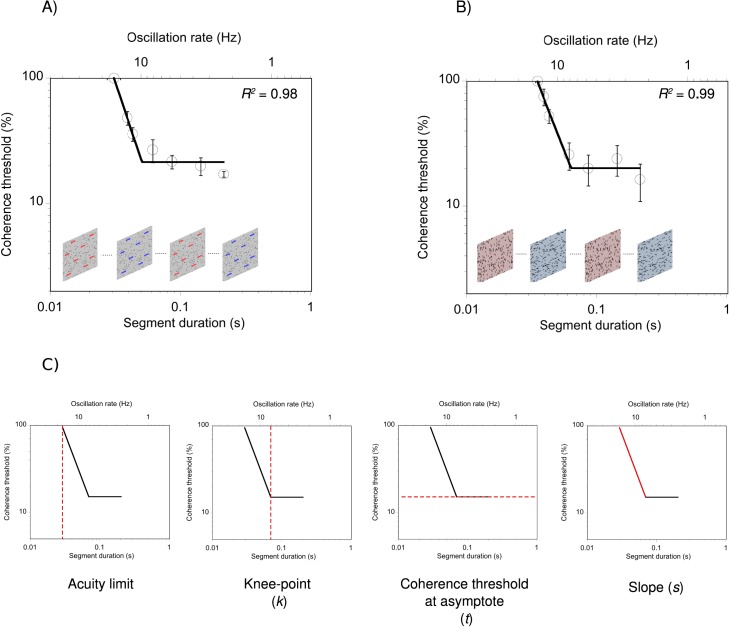
Representative data for a single participant on (A) the motion task and (B) the form task. The dashed red line in (C) represents the measured temporal acuity limit together with the three best-fitting parameters (*k, t,* and *s)* from Equation 1. Error bars = ±1 *SEM*.

To quantify the relationship between segment duration and perceptual performance we fitted a two-limbed curve (Equation 1) to each participant's data using a conventional least-squares fitting procedure, the Levenberg–Marquardt algorithm. This curve has previously been used to characterize and quantify performance on motion tasks (Allen, Hutchinson, Ledgeway, & Gayle, 2010; Hutchinson & Ledgeway, 2010; Hutchinson, Ledgeway, Allen, Long, & Arena, 2013; Johnston et al., 2016b):

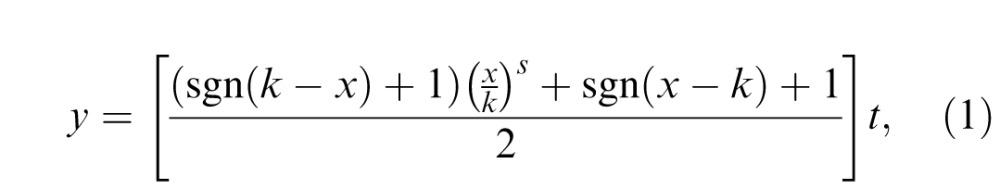
where *x* is segment duration, and *k*, *t*, and *s* are free parameters. Parameter *k* is the knee-point of the function and represents the segment duration above which performance no longer improves. Parameter *t* is the coherence threshold at asymptote, while parameter *s* is the slope of the descending limb of the curve. Sgn(), the signum function, is equal to either −1, 0, or +1 depending on whether the argument in parentheses is <0, 0, or >0, respectively. In all cases, the data were well described by the function (mean *R*^2^ for the motion task = 0.94, *SD* = 0.05, range = 0.79 to 0.99; mean *R*^2^ for the form task = 0.95, *SD* = 0.04, range = 0.82 to 0.99). Representative data for a single participant on the motion task and the form task is shown in Figure 2A and 2B, respectively. The empirically measured acuity limit together with the three best-fitting parameters derived from the curve-fit procedure (knee-point, coherence threshold at asymptote, and slope) are depicted in Figure 2C.

### Statistical analyses

Developmental dyslexia has primarily been associated with poor phonemic decoding skills (Snowling, 2000). However, evidence suggests that the pattern of performance found on low-level visual perception tasks, requiring the processing of motion and form, cannot differentiate generally poor readers from individuals with poor phonemic decoding skills, consistent with the dyslexic profile (Hulslander et al., 2004; Johnston et al., 2016a, 2016b; Talcott et al., 1998). We took advantage of this finding in the present study and conducted a series of continuous analyses, using a composite measure of reading skill. First, scores for the reading tests were *z*-transformed to allow comparisons between different scores. Bivariate correlations (Pearson's product-moment correlation coefficient) were then used to investigate the relationships between the individual measures of reading ability. If correlations are strong, principal component analysis (PCA) can be used to reduce the dimensionality of the data and isolate a single construct of common variation among the three reading tests (Johnston et al., 2016a, 2016b; Pugh et al., 2013, 2014).

In addition, as previous research has shown that gender and nonverbal IQ are associated with performance on some motion tasks (Johnston et al., 2016a, 2016b; Melnick, Harrison, Park, Bennetto, & Tadin, 2013; Snowdon & Kavanagh, 2006), we conducted a series of semipartial correlations to investigate if composite reading scores explained any additional variance in task performance after controlling for the effects of gender and nonverbal IQ. This is especially important in the current study, as considerably more females than males participated. Some of the best-fitting parameters for the motion task (acuity limits, the knee-point of the curves, and the slope of the descending limb of the curves) and the form task (the knee-point of the curves, and the slope of the descending limb of the curves) violated the assumption of normality (Shapiro–Wilk test, *p* < 0.03). Thus, a series of nonparametric, semipartial correlations were performed on these variables using Spearman's rank-order correlation coefficient (*r_s_*). Acuity limits for the motion task and coherence thresholds at asymptote for both tasks did not violate the assumption of normality (Shapiro–Wilk test, *p* > 0.08) so a series of parametric, semipartial correlations were performed on these variables using Pearson's product-moment correlation coefficient (*r*).

## Results

### Principal component analyses: Composite reading score

**Figure 3:**
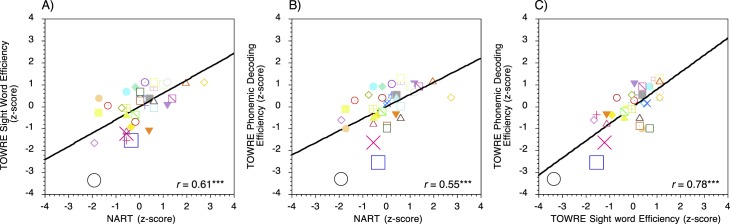
Scatter plots showing the relationships between scores on the three individual measures of reading ability. Positive and negative *z*-scores indicate performance that is better or worse than the mean of the sample. Three participants (indicated by the larger symbols) had standard scores ≤85 (at or below the 15th percentile) on the TOWRE Phonemic Decoding subtest, which falls into the conventional range for identifying individuals with developmental dyslexia (Heath, Bishop, Hogben, & Roach, 2006; Johnston et al., 2016a; Pugh et al., 2014). NART = National Adult Reading Test; TOWRE = Test of Word Reading Efficiency. * = *p* < 0.05; ** = *p* < 0.01; *** = *p* < 0.001.

Scatters plots showing the relationships between scores on the three individual measures of reading ability are shown in Figure 3. The correlations between the individual measures of reading ability were significant and strong (*r* = 0.55–0.78, *p* < 0.001) so PCA was conducted to calculate the composite measure of reading skill. Raw scores for the three reading tests were entered into the analysis, which was performed on the correlation matrix. A single principal component accounted for 77% of the total variance among the individual measures of reading ability (eigenvalue 1 = 2.30; eigenvalue 2 = 0.49; eigenvalue 3 = 0.22). Loadings for the TOWRE Sight Word Efficiency subtest and the TOWRE Phonemic Decoding Efficiency subtest were within the same range (0.92 and 0.90, respectively), but the NART contributed slightly less (loading = 0.81). PCA scores for each individual were entered into the whole-sample analyses to investigate how general reading ability relates to performance on the motion and form tasks.

### Motion task: Semipartial correlations

**Figure 4:**
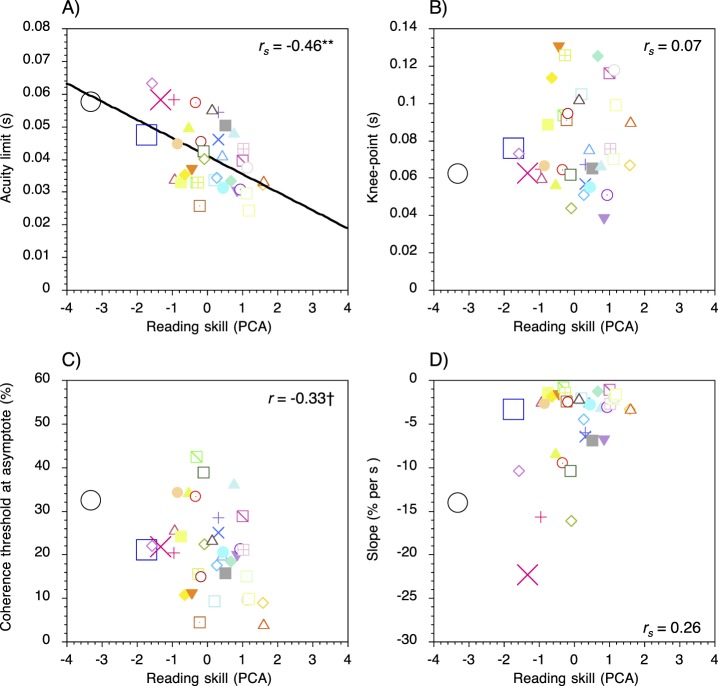
Scatter plots for the motion task showing the relationships between (A) reading ability and temporal acuity limits, (B) the knee-point of the curves, (C) coherence thresholds at asymptote, and (D) the slope of the descending limb. Each colored symbol represents an individual participant. Positive and negative scores on the composite measure of reading skill indicate performance that is better or worse than the mean of the sample, respectively. The three participants with the lowest standard scores (≤ 85) on the TOWRE Phonemic Decoding subtest are indicated by the larger symbols. *Note*. † Nonsignificant trend; * *p* < 0.05; ** *p* < 0.01; *** *p* < 0.001.

Scatter plots for the motion task displaying the relationships between reading ability and acuity limits and reading ability and each of the three curve-fit parameters are shown in Figure 4. Scores on the composite measure of skill were significantly and negatively correlated with temporal acuity limits on the motion task, *r_s_* = −0.46*, p* < 0.01. The minimum segment duration needed to detect the test stimulus was longer in relatively poor readers (i.e., those with lower composite scores for reading) than relatively good readers (i.e., those with higher composite scores for reading). General reading ability was not significantly correlated with the knee-point of the curves, *r_s_* = 0.07*, p* = 0.68. However, a nonsignificant trend was found between composite reading scores and coherence thresholds at asymptote, *r* = −0.33*, p* = 0.051. The point on the *y*-axis (Figure 2C) at which performance became asymptotic was lower in relatively poor readers than relatively good readers. Reading ability was not significantly correlated with the slope of the descending limb of the curves, *r_s_* = 0.26*, p* = 0.12.

### Form tasks: Semipartial correlations

**Figure 5:**
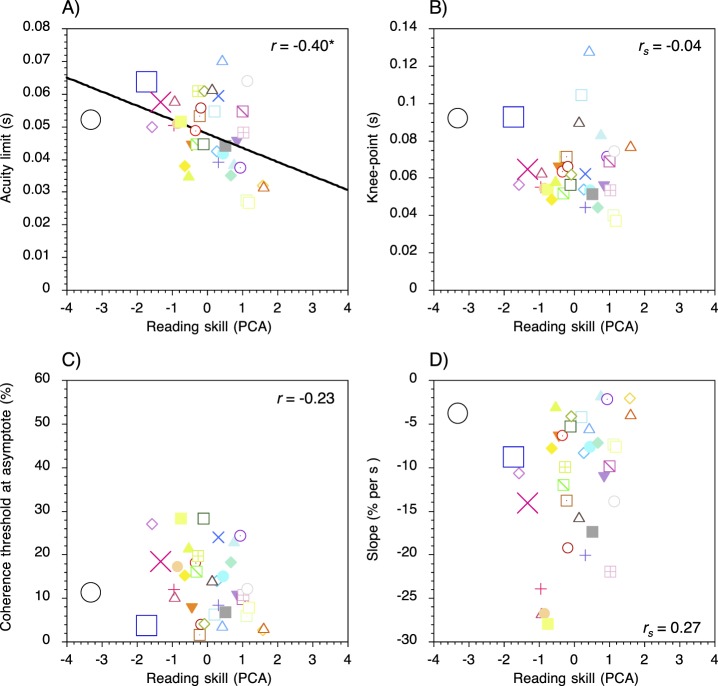
Scatter plots for the form task showing the relationships between (A) reading ability and temporal acuity limits, (B), the knee-point of the curves, (C) coherence thresholds at asymptote, and (D) and the slope of the descending limb. Each colored symbol represents an individual participant. Positive and negative scores on the composite measure of reading skill indicate performance that is better or worse than the mean of the sample, respectively. The three participants with the lowest standard scores (≤85) on the TOWRE Phonemic Decoding subtest are indicated by the larger symbols.* *p* < 0.05; ** *p* < 0.01; *** *p* < 0.001.

Scatter plots for the form task displaying the relationships between reading ability and acuity limits and reading ability and each of the three curve-fit parameters are shown in Figure 5. Scores on the composite measure of skill were significantly and negatively correlated with temporal acuity limits on the form task, *r* = −0.40*, p* = 0.02. The minimum segment duration needed to detect the test stimulus was longer in relatively poor readers than relatively good readers. No significant correlation was found between reading ability and the knee-point of the curves, *r_s_* = −0.04*, p* = 0.83, coherence thresholds at asymptote, *r* = −0.23*, p* = 0.18, nor the slope of the descending limb of the curves, *r_s_* = 0.27*, p* = 0.11.

## Discussion

Evidence suggests that adult poor readers have difficulty segmenting local motion cues across space (Johnston et al., 2016b). However, the effects of reading ability on temporal segmentation are currently unclear as previous work has relied on a single measurement of sensitivity and has failed to delineate temporal segmentation of motion and form information (Talcott et al., 1998). Here, we systematically addressed these limitations by administering a new set of motion and form tasks that were temporal versions of spatial segmentation tasks previously used to investigate the visual deficit in dyslexia (Johnston et al., 2016b). Results showed that temporal acuity limits for the motion and form tasks were significantly and negatively correlated with scores on the composite measure of reading skill, consistent with previous research that has explored the effects of reading ability on CFF thresholds (Holloway, Náñez, & Seitz, 2013; Talcott et al., 1998). The minimum segment duration needed to detect the test stimulus was longer in relatively poor readers than relatively good readers. However, our results extend previous findings in two key ways. First, our results suggest that relatively poor readers have a generic difficulty with temporal segmentation that transcends domains as a similar pattern of results (both strength and significance of correlation) was found for both the motion and form tasks. Second, our results reveal that the difficulties with temporal segmentation of motion and form information shown by relatively poor readers are pronounced at short segment durations.

The results of the current study cast further doubt on the notion that adult poor readers have a selective impairment in the dorsal visual processing stream as temporal acuity limits for the motion and form tasks were significantly correlated with general reading ability. These results corroborate those of Johnston et al. (2016a, 2016b) and challenge the dorsal stream vulnerability hypothesis of developmental dyslexia. Braddick et al. (2016) have suggested that visual deficits found in generally poor readers are qualitatively distinct to those observed in developmental dyslexia. However, other studies have shown that visual difficulties cannot differentiate generally poor readers from individuals with impaired phonological decoding skills consistent with the dyslexic profile (Hulslander et al., 2004; Johnston et al., 2016a, 2016b; Talcott et al., 1998). In the current study, three participants met the conventional criterion for developmental dyslexia (≤85 on the TOWRE Phonemic Decoding Efficiency subtest) but they were indistinguishable from generally poor readers on the basis of their visual performance, with the exception of one reader with dyslexia who had the shallowest slope parameter on the motion task (Figure 4D). Further research is needed to address the reliability of this finding but at present, it is sufficient to conclude that performance on low-level visual perception tasks, requiring the processing of motion and form, cannot typically differentiate adult poor readers from individuals with developmental dyslexia.

Our findings are consistent with the notion that adult poor readers have difficulty on visual tasks requiring rapid encoding of temporal information in the brain. In our study, the stimuli in the motion task consisted of individual dots that were displaced on each frame to create the perception of apparent motion, whereas the stimuli in the form task comprised directly analogous dot streaks (Johnston et al., 2016a, 2016b; Simmers et al., 2005). Although both tasks necessitated segmentation of local visual cues over time, the motion task required a finer, additional scale of temporal processing than the form task because the positions of local dots was constantly being updated. The correlation between temporal acuity limits and scores on the composite measure of reading skill was slightly stronger for the motion task than the form task. As adult poor readers also exhibit impaired performance on auditory tasks containing acoustic signals that are constantly changing over time, this implies a generic deficit with the parsing of time-varying information that extends to other sensory modalities (Giraud & Poeppel, 2012; Goswami, 2011, 2016; Lehongre et al., 2013; Lehongre, Ramus, Villiermet, Schwartz, & Giraud, 2011; Pammer, 2014).

Recent work has suggested that spatial scale selection is impaired in generally poor readers and individuals with dyslexia (Johnston et al., 2016b). To reliably perform the motion and form tasks in the present study, local visual cues had to be integrated over a certain period of time. Our results are not indicative of a deficit with temporal scale selection, as the knee-point and slope of the descending limb of the curves for the motion and form tasks was not significantly associated with general reading skill. Thus, the underlying nature of the visual deficit in adult poor readers appears to have at least two distinct components, one spatial and one temporal; that is, a difficulty in selecting the spatial scale optimal for performance and an insensitivity to visual stimuli that change over time, particularly when that temporal change is rapid.

A deficit in temporal acuity could also explain why reading ability is significantly correlated with coherence thresholds on conventional RDK tasks but not global form tasks, as relatively poor readers could sample individual frames of the motion sequence at a lower rate. However, under certain conditions, reduced temporal sampling can have a facilitatory effect. Research has shown that RDK tasks cannot be reliably performed when a delay greater than approximately 100 ms is introduced between consecutive frames (Baker & Braddick, 1985). Mather and Tunley (1995) found that larger interframe intervals could be tolerated when low-pass temporal filtering was applied to the motion sequence in order to reduce sampling rate. Hence, it should be possible to simulate conditions under which relatively poor readers outperform relatively good readers on RDKs tasks (i.e., when a relatively long temporal delay is introduced between consecutive frames).

Most research has conducted between-groups analyses to compare visual and neurocognitive function in adults with and without dyslexia, whereas in the present study, a series of continuous analyses were performed using a composite measure of reading skill (Johnston et al., 2016b; Pugh et al., 2013, 2014). Between-groups analyses are more likely to suffer from low statistical power than continuous analyses because sample size is effectively halved in the former, relative to the latter. This has been identified as a contributing factor to the lack of reproducibility in biomedical research (Button et al., 2013; Loannidis, 2005; Munafò et al., 2017). In addition, reading difficulties are notoriously difficult to define (Fletcher, 2009). Hence, a wide range of criteria has been used to identify individuals with dyslexia, which might also contribute to inconsistencies across studies. The finding that visual difficulties cannot generally differentiate poor readers from individuals with dyslexia justifies the use of continuous analyses in future research. Adopting these types of experimental design will improve the reliability of scientific research as they enhance statistical power and do not require decisions to be made regarding controversial definitional criteria and arbitrary cut-offs.

In the present study, we conducted semipartial correlations to investigate if composite reading scores predicted task performance after controlling for gender and nonverbal IQ, which are known to be associated with motion perception (Arranz-Paraíso & Serrano-Pedrazza, 2016; Cook, Hammett, & Larsson, 2016; Johnston et al., 2016a; Melnick et al., 2013). The very limited number of male participants in the current study (6/38) precludes a meaningful investigation of how gender relates to visual performance. However, additional analyses across the whole sample revealed that nonverbal IQ was significantly and negatively correlated with coherence thresholds at asymptote on both the motion task (*r* = −0.46*, p <* 0.01) and the form task (*r* = −0.48*, p <* 0.01), after partialling out reading ability and gender. Individuals with a lower IQ had reduced sensitivity at asymptote than individuals with a higher IQ. These results extend previous findings by showing that nonverbal IQ is also associated with tasks in which either motion or form cues need to be segmented over time. Why nonverbal IQ is significantly associated with performance on some psychophysical tasks is currently unclear. Nonetheless, our results highlight the critical importance of controlling for nonverbal IQ when exploring visual performance in dyslexia and other neurodevelopmental disorders.

## Conclusions

In summary, we found that adult poor readers have difficulty segmenting temporally changing motion and form cues, particularly at short segment durations. This pattern of results is consistent with a growing body of research suggesting that rapid encoding of time-varying information is impaired in adults with developmental dyslexia (Johnston et al., 2016a, 2016b).
